# Surgery After BRAF-Directed Therapy Is Associated with Improved Survival in BRAF^V600E^ Mutant Anaplastic Thyroid Cancer: A Single-Center Retrospective Cohort Study

**DOI:** 10.1089/thy.2022.0504

**Published:** 2023-04-10

**Authors:** Xiao Zhao, Jennifer Rui Wang, Ramona Dadu, Naifa Lamki Busaidy, Lei Xu, Kim O. Learned, Noah N. Chasen, Thinh Vu, Anastasios Maniakas, Arturo A. Eguia, Julia Diersing, Neil D. Gross, Ryan Goepfert, Stephen Y. Lai, Marie-Claude Hofmann, Renata Ferrarotto, Charles Lu, Gary Brandon Gunn, Michael T. Spiotto, Vivek Subbiah, Michelle D. Williams, Maria E. Cabanillas, Mark E. Zafereo

**Affiliations:** ^1^ Department of Head and Neck Surgery; Houston, Texas, USA.; ^2^ Endocrine Neoplasia and Hormonal Disorders; Houston, Texas, USA.; ^3^ Department of Neuroradiology; Houston, Texas, USA.; ^4^ Thoracic-Head and Neck Medical Oncology; Houston, Texas, USA.; ^5^ Radiation Oncology; Houston, Texas, USA.; ^6^ Investigational Cancer Therapeutics; Houston, Texas, USA.; ^7^ Pathology; The University of Texas MD Anderson Cancer Center, Houston, Texas, USA.

**Keywords:** anaplastic thyroid cancer, thyroid cancer, BRAF^V600E^, BRAF therapy, neoadjuvant, surgery

## Abstract

**Background:**

The aim of this study was to describe the oncologic outcomes of patients with BRAF^V600E^-mutated anaplastic thyroid cancer (ATC) who had neoadjuvant BRAF-directed therapy with subsequent surgery. For context, we also reviewed patients who received BRAF-directed therapy after surgery, and those who did not have surgery after BRAF-directed therapy.

**Methods:**

This was a single-center retrospective cohort study conducted at a tertiary care cancer center in Texas from 2017 to 2021. Fifty-seven consecutive patients with BRAF^V600E^-mutated ATC and at least 1 month of BRAF-directed therapy were included. Primary outcomes were overall survival (OS) and progression-free survival (PFS).

**Results:**

All patients had stage IVB (35%) or IVC (65%) ATC. Approximately 70% of patients treated with BRAF-directed therapy ultimately had surgical resection of residual disease. Patients who had neoadjuvant BRAF-directed therapy followed by surgery (*n* = 32) had 12-month OS of 93.6% [confidence interval (CI) 84.9–100] and PFS of 84.4% [CI 71.8–96.7]. Patients who had surgery before BRAF-directed therapy (*n* = 12) had 12-month OS of 74.1% [CI 48.7–99.5] and PFS of 50% [CI 21.7–78.3]. Finally, patients who did not receive surgery after BRAF-directed therapy (*n* = 13) had 12-month OS of 38.5% [CI 12.1–64.9] and PFS of 15.4% [CI 0–35.0]. Neoadjuvant BRAF-directed therapy reduced tumor size, extent of surgery, and surgical morbidity score. Subgroup analysis suggested that any residual ATC in the surgical specimen was associated with significantly worse 12-month OS and PFS (OS = 83.3% [CI 62.6–100], PFS = 61.5% [CI 35.1–88]) compared with patients with pathologic ATC complete response (OS = 100%, PFS = 100%).

**Conclusions:**

We observed that neoadjuvant BRAF-directed therapy reduced extent of surgery and surgical morbidity. While acknowledging potential selection bias, the 12-month OS rate appeared higher in patients who had BRAF-directed therapy followed by surgery as compared with BRAF-directed therapy without surgery; yet, it was not significantly different from surgery followed by BRAF-directed therapy. PFS appeared higher in patients treated with neoadjuvant BRAF-directed therapy relative to patients in the other groups. These promising results of neoadjuvant BRAF-directed therapy followed by surgery for BRAF-mutated ATC should be confirmed in prospective clinical trials.

## Introduction

Anaplastic thyroid cancer (ATC) accounts for ∼1% of thyroid cancers but causes ∼50% of thyroid cancer mortality.^1,2^ Before the last decade, 1-year survival had been reported at ∼20% with a median survival of ∼5 months.^1^ While surgery is considered first-line treatment for patients presenting with resectable disease, the vast majority of patients present with disease that is not meaningfully resectable owing to carotid/innominate artery encasement, larygnotracheoesophageal involvement, and/or significant mediastinal or distant disease.^3,4^ In addition, these surgeries are often associated with gross residual disease. Induction cytotoxic chemotherapy has been studied to reduce tumor volume and facilitate surgery; however, response rates are low, ∼20–30%.^5,6^

Over the last 5 years, molecular targeted therapy has dramatically changed the treatment paradigm for ATC. Approximately 40% of patients with ATC have a targetable BRAF^V600E^ mutation.^2^ BRAF^V600E^ mutation results in constitutive activation of BRAF and the downstream MAPK pathway, a major oncogenic pathway for cancer initiation and progression.^7^ Treatment of BRAF^V600E^-mutated ATC with a combination of BRAF and MEK inhibitors has resulted in dramatic response rates that are substantially improved from chemoradiation.^8^ As such, patients with BRAF^V600E^-mutated ATC on a BRAF/MEK inhibitor have a median overall survival (OS) of 14.5 months.^9,10^

BRAF/MEK inhibition is now standard of care for locally advanced or metastatic BRAF^V600E^ mutant positive ATC. Yet, even with the response to BRAF inhibitors, very few patients achieve complete response (CR), with the majority of patients having residual disease.^2,10^ In addition, through long-term use of BRAF inhibitors, it is now well recognized that most BRAF^V600E^-mutated tumors ultimately develop resistance, such as the acquisition of a secondary RAS/RAF mutation or overexpression of membrane receptors that reactivate the MAPK pathway.^7,11–13^

Taken together, despite the dramatic effect of BRAF inhibition, the high likelihood of residual disease and development of resistance demonstrates a need for consolidative therapies, such as surgery, to augment BRAF-directed therapy. We performed a retrospective cohort study of BRAF^V600E^-mutated ATC patients treated with BRAF-directed therapy at MD Anderson Cancer Center (MDACC) to explore the utility of surgery following neoadjuvant BRAF/MEK inhibitor treatment and its potential effect on overall and progression-free survival (PFS).

## Methods

### Study population and demographics

This single-center retrospective cohort study was approved by the MDACC Institutional Review Board (PA14-1082). We included consecutive patients between January 2017 and December 2021 with a diagnosis of BRAF^V600E^-mutated ATC who were treated with BRAF-directed therapy for at least 1 month. BRAF-directed therapy was defined as treatment with a BRAF and MEK inhibitor with or without checkpoint inhibitor immunotherapy. BRAF inhibitors utilized in this study included: vemurafenib, dabrafenib, and encorafenib. MEK inhibitors include cobimetinib, trametinib, and binimetinib. Immunotherapy was defined as any treatment with a PD-1 or PDL-1 checkpoint inhibitor. Patients with ATC were excluded if they did not meet the inclusion criteria or if they were lost to follow-up. For a patient on neoadjuvant BRAF-directed therapy, BRAF inhibitor is stopped 1 day before surgery and MEK inhibitor is stopped 3–5 days before surgery. Both BRAF and MEK inhibitors are started 5–7 days postoperatively. Demographics, imaging analysis of CT and PET-CT scans, pathology, and survival data were extracted from electronic medical records. TNM classification and staging was performed using the American Joint Committee on Cancer Classification, 8th Edition. Diagnosis of ATC was performed based on histopathologic assessment of biopsy or surgical specimen by a head and neck pathologist. BRAF^V600E^ mutation was determined by next-generation sequencing (NGS) of tumor tissue and/or blood (liquid biopsy) and/or by immunohistochemistry (IHC).

### Study groups

Patients were divided into three groups: (1) neoadjuvant + surgery: defined as patients who received definitive surgery for primary site disease after neoadjuvant BRAF-directed therapy (*n* = 32 patients). (2) No surgery defined as patients who did not receive surgery after initiating BRAF-directed therapy (*n* = 13 patients). (3) Upfront surgery: defined as patients who received definitive surgery for primary site disease before starting BRAF-directed therapy (*n* = 12 patients). Definitive surgery was defined as intent for total or near total resection of thyroid disease in the neck.

### Surgery and Thyroid Neck Morbidity and Complexity Scoring System

To quantify the complexity of surgery and its associated morbidity, the Thyroid Neck Morbidity and Complexity (TNMC) scoring system was used (Supplementary Table S2). Since the study of how neoadjuvant therapy may change surgical morbidity in patients with advanced thyroid cancer is a very new concept, there unfortunately is not yet a validated scoring system for this purpose. The TNMC scoring system, developed for several multicenter advanced thyroid cancer neoadjuvant clinical trials, is an unvalidated scoring system designed to objectify the effect of neoadjuvant treatment on surgical morbidity for advanced thyroid cancer. TNMC scoring pre-BRAF–directed therapy was determined by assessing the head and neck CT scan performed at initial presentation. TNMC score post-BRAF therapy was assessed based on the intraoperative experience of the surgeon as per the operative report.

### Pathology assessment

Initial pathologic classification for BRAF^V600E^ status was performed on biopsy specimens of all included ATC patients. The diagnosis was confirmed by IHC alone (*n* = 7/57), NGS alone (*n* = 7/57), or both (*n* = 43/57). Of the patients who had both modalities, two patients had equivocal BRAF status by IHC, which was confirmed by NGS. The surgical specimen diagnosis of ATC and/or papillary thyroid cancer (PTC) in the neoadjuvant setting was performed by one of the three dedicated head and neck pathologists, each with at least 18 years of experience. Categorization of residual ATC and/or PTC was determined by standard hematoxylin and eosin–stained slide assessment of the entire surgical specimen with a focus on the primary site specimen and lymph node metastasis if present. Pathologic ATC CR was defined as having no ATC in the surgical specimen with or without viable PTC.

### CT assessment

Analysis of change in tumor size was performed by two head and neck radiologists (K.O.L. and T.V.) with 13–15 years of experience and any discrepancies were resolved in consensus. On CT scan, the ATC tumor was measured by the longest dimension to obtain the lesion diameter. Response assessment was assessed by absolute change in diameter size and by percentage change in diameter through comparison of initial and preoperative CT scans, following RECIST criteria.^14^ Initial CT scan was defined as the most recent CT scan performed before initiating BRAF-directed therapy. Preoperative CT scan was defined as the most recent CT scan obtained before having definitive surgery.

### Adverse events and surgical complications

For the neoadjuvant + surgery group, major adverse events during the neoadjuvant period before surgery were reviewed and categorized. Major adverse events were defined as those associated with hospitalization, drug hold, and/or drug dose modification. Similarly, for the neoadjuvant + surgery group, surgical complications, including return to the operating room or hospital within 30 days, were reviewed and categorized.

### Statistical analysis

Demographic and treatment characteristics of the study groups were presented as mean with standard deviation and median with interquartile range (IQR) for continuous variables of normal and non-normal distribution, respectively, and as number with percentage for categorical data. OS was measured from date of diagnosis of ATC to date of death, with patients censored at last follow-up. PFS was measured from date of diagnosis to date of structural progression. The Kaplan–Meier method was used to calculate survival rates and corresponding confidence intervals. Kaplan–Meier survival curves for OS and PFS were plotted and compared using the log-rank test. In the subgroup analysis in patients of neoadjuvant + surgery group, univariate Cox proportional hazards regression followed by multivariate Cox regression analyses were performed to compare OS and PFS based on stage at diagnosis, preoperative TNMC score, and time from initiating BRAF-directed therapy to surgery. Age and stage at diagnosis were included in multivariate analyses because they are known prognostic factors for ATC. A two-sided *p* < 0.05 indicated statistical significance. SAS 9.4 was used to perform survival analysis. Graphical data were plotted using GraphPad Prism 9.0.

## Results

**FIG. 1.**
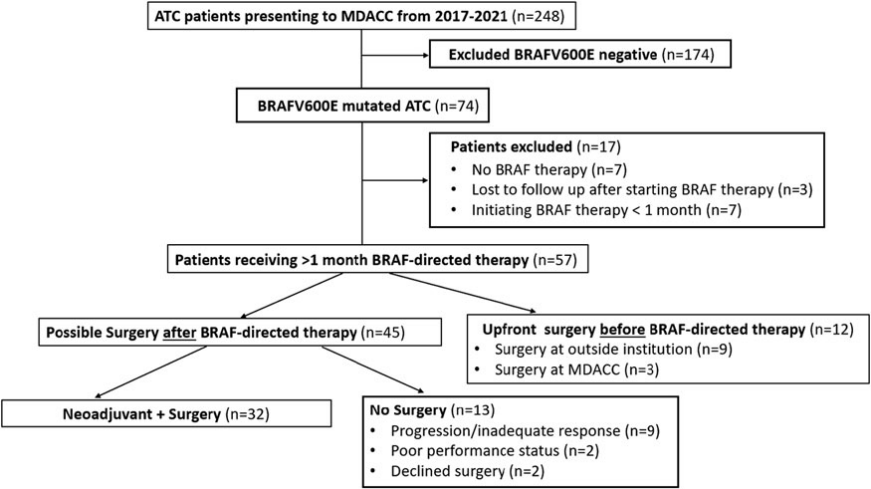
Flow diagram for study cohorts. ATC, anaplastic thyroid cancer; MDACC, MD Anderson Cancer Center.

From 2017 to 2021, 248 patients presented to MDACC with ATC (Fig. 1). Of these patients, a total of 57 patients with BRAF^V600E^-mutated ATC with more than 1 month of BRAF-directed therapy were included in this study. This cohort was divided into three groups (1) neoadjuvant + surgery group: defined as definitive surgery performed after BRAF-directed therapy, (2) no surgery group: defined as patients who did not receive surgery after BRAF-directed therapy, and (3) upfront surgery group: defined as patients who had definitive surgery before BRAF-directed therapy. Demographics of these three groups are given in Table S1. Patients in the neoadjuvant + surgery group were younger versus the other groups (analysis of variance [ANOVA], *p* = 0.04) (Supplementary Table S1). There were significantly more patients in the upfront surgery group who received postoperative radiation therapy (92%) compared with the neoadjuvant + surgery group (50%) (Fisher's exact test, *p* = 0.015). Of interest, there were no stage IVA patients and the majority presented with distant metastasis [stage IVB (35%) vs. IVC (65%)].

**Table 1.** Demographics and Clinical Characteristics for BRAF^V600E^ anaplastic Thyroid Cancer Patients Treated with BRAF-Directed Therapy

	Neoadjuvant + surgery (*n* = 32)	No surgery (*n* = 13)	Upfront surgery (*n* = 12)
Age	61.5 ± 9.3	67.2 ± 11.3	68.6 ± 6.0
Sex, *n* (%)			
Male	15 (46.9)	8 (61.5)	7 (58.3)
Female	17 (53.1)	5 (38.5)	5 (41.7)
AJCC 8th edition stage, *n* (%)			
IVA	0	0	0
IVB	12 (37.5)	2 (15.4)	6 (50.0)
IVC	20 (62.5)	11 (84.6)	6 (50.0)
Treatment, *n* (%)			
Immunotherapy	27 (84.4)	9 (69.2)	7 (58.3)
Post-op XRT	16 (50.0)	NA	11 (91.7)

Age represented by mean and standard deviation.

AJCC, American Joint Committee on Cancer; XRT, radiation therapy.

For patients in the neoadjuvant + surgery group (*n* = 32), median follow-up was 35.7 months (IQR, 27.6–46.9 months). Median time from initiation of BRAF-directed therapy to surgery was 136 days (IQR, 77–492 days). Fourteen (44%) of 32 patients had adverse events, which generally required a temporary drug hold and/or dose modification during the neoadjuvant period and 4 (13%) patients in the neoadjuvant + surgery group had surgical complications (Supplementary Table S3).

For patients in the no surgery group (*n* = 13), median follow-up was 33.2 months (IQR, 27.2–39.1 months). Of these patients, nine patients had an inadequate response with either immediate progression, short duration of response, or mixed response (primary vs. metastasis), which precluded surgery. Two patients were not offered surgery because of poor performance status and two patients declined surgery. Finally, for patients in the upfront surgery group (*n* = 12), median follow-up was 47.9 months (IQR, 33.6–63.9 months). In this group, nine patients had surgery at outside institutions before assessment at MDACC and the remaining three patients had surgery at MDACC. Of these three patients, two did not have a diagnosis of ATC before surgery, and the final patient had surgery at MDACC in 2017 before the establishment of the neoadjuvant paradigm for BRAF-mutated ATC.

**FIG. 2.**
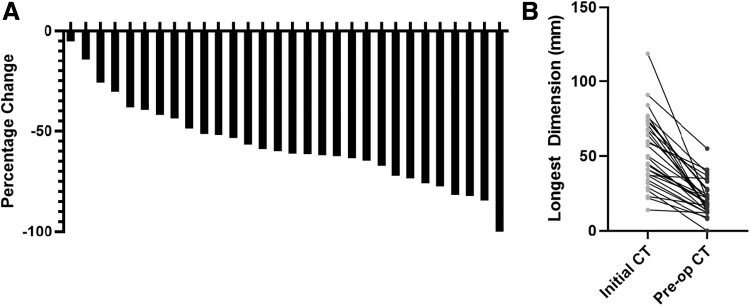
Response to BRAF-directed therapy by CT imaging. (**A**) Percent change in tumor by longest axis by CT imaging after BRAF-directed therapy. (**B**) Absolute size change in tumor size as measured by longest axis. (*n* = 30).

**FIG. 3.**
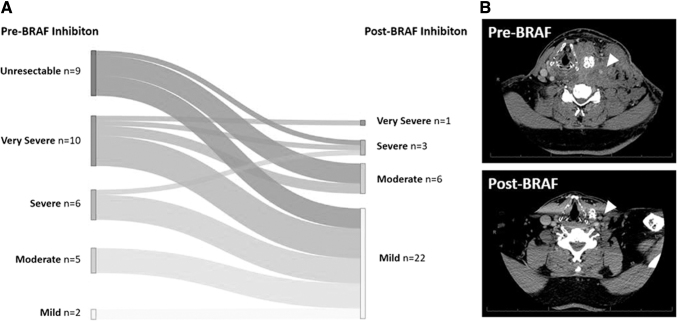
Change in surgical morbidity after BRAF-directed therapy. (**A**) Sankey graph demonstrating changes in surgical morbidity before BRAF-directed therapy (left) and after BRAF-directed therapy (right) as measured using the TNMC scoring system. (**B**) Example of patient presenting as initially inoperable owing to common carotid encasement and regression of tumor from carotid after BRAF therapy. TNMC, Thyroid Neck Morbidity and Complexity.

Approximately 70% of eligible candidates for definitive surgery after BRAF-directed therapy (32/45 patients) went on to receive surgery (Fig. 1). For these patients, BRAF-directed therapy offered a marked reduction in tumor size and extent of surgical resection. The pretreatment and preoperative CT scans were compared to estimate the tumor size reduction. The diameter of the largest neck lesion was reduced by a median of 61% (IQR, 44–72) (Fig. 2A). The largest neck lesion decreased from a median of 50 mm (IQR, 37–61 mm) to 19 mm (IQR, 13–27 mm) (Fig. 2B). This reduction in tumor size after BRAF-directed therapy corresponded to a reduction in extent of surgery and surgical morbidity. To quantify the complexity of surgery and its associated morbidity, the TNMC scoring system was used (Supplementary Table S2). Nine patients (28%) had unresectable disease at initial presentation, primarily because of common carotid/innominate encasement and/or prevertebral fascia involvement (Fig. 3). In addition, 50% of patients (*n* = 16/32) initially had a TNMC classification of very severe or severe morbidity (expected to require extended surgery including but not limited to a laryngopharyngectomy, esophagectomy, and/or tracheal resection). In contrast, after BRAF-directed therapy, only one patient required a laryngectomy and one patient received a tracheal resection. Most patients had a reduction in TNMC score to mild morbidity (69%, *n* = 22/32). Specifically, of the nine patients who were initially classified as unresectable, all but one patient were recategorized as moderate or mild surgical morbidity. As resection of ATC postneoadjuvant therapy is challenging, primarily owing to significant desmoplastic tissue reaction and fibrosis, a thyroid lobectomy (rather than total thyroidectomy) was chosen in eight patients with unilateral disease to ensure the contralateral recurrent laryngeal nerve was not threatened.

**FIG. 4.**
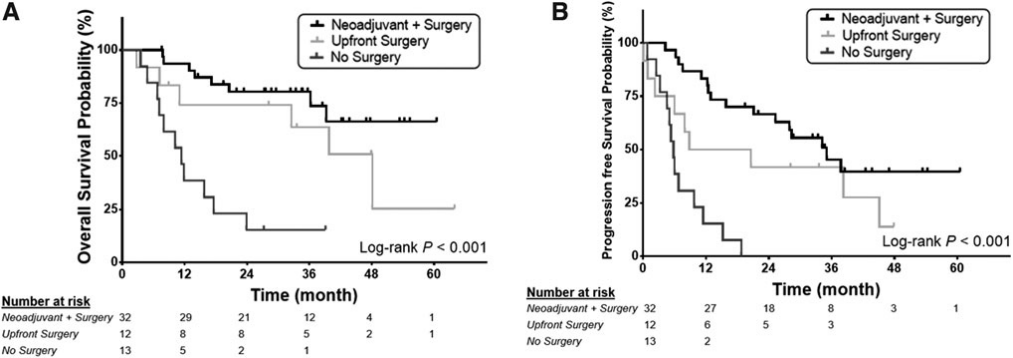
OS and PFS for patients in relation to surgery after BRAF-directed therapy. Red neoadjuvant + surgery group: Patients who had surgery after BRAF-directed therapy, Green: upfront surgery group: Patients who had surgery before BRAF-directed therapy, Blue: No surgery group: Patients who received BRAF-directed therapy but did not receive surgery. (**A**) OS. (**B**) PFS. OS, overall survival; PFS, progression-free survival.

For patients in the neoadjuvant + surgery group, median OS was not reached [CI 39.2–NA months] and PFS was 34.2 months [CI 15.8–NA months] (Fig. 4A, B). The 12- and 24-month OS was 93.6% [CI 84.9–100] and 80.3% [CI 66.1–94.5]. The 12- and 24-month PFS was 84.4% [CI 71.8–96.7] and 62.2% [CI 45.3–79.1]. For patients in the no surgery group, median OS and PFS was 11.4 months [CI 6.7–17.6 months] and 5.8 months [CI 3.2–9.8 months], respectively (Fig. 4A, B). The 12- and 24-month OS was 38.5% (12.1–64.9%) and 15.4% [CI 0–35]. The 12- and 24-month PFS was 15.4% (0–35.0%) and 0%. Finally, for patients who had upfront surgery, median OS and PFS was 48.1 months [CI 7.1–NA months] and 14.7 months [CI 0.9–45.1 months], respectively. The 12- and 24-month OS was 74.1% [CI 48.7–99.5] and 74.1% [CI 48.7–99.5]. The 12- and 24-month PFS was 50% (21.7–78.3%) and 41.7% [CI 13.8–69.6]. Given the significant difference in age between the three groups (Supplementary Table S1), we performed a multivariate Cox proportional hazards analysis adjusted for age and stage. Age and stage were not significant predictive factors associated with OS or PFS.

**FIG. 5.**
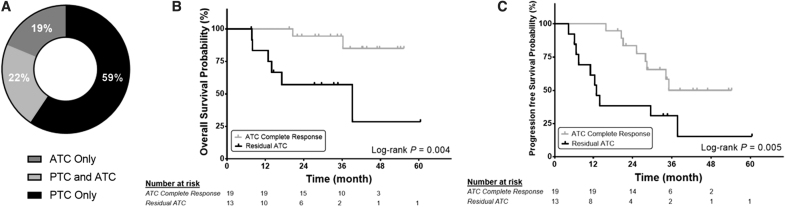
OS and PFS based on surgical pathology. (**A**) Pathology of surgical specimens separated based on PTC and ATC. (**B**) OS and (**C**) PFS based for pathologic residual ATC or ATC CR after definitive surgery. CR, complete response; PTC, papillary thyroid cancer.

Subgroup analysis for patients in the neoadjuvant + surgery group demonstrated that stage at diagnosis, preoperative TNMC score, and time from initiating BRAF-directed therapy to surgery did not impact OS or PFS using a Cox proportional hazard regression model (Supplementary Table S4). Final surgical pathology was significantly associated with outcomes. About 59% (*n* = 19/32) of patients had a pathologic ATC CR with only PTC remaining in their surgical specimen (Fig. 5A). These patients had significantly improved OS (Fig. 5B) and PFS (Fig. 5C) compared with patients with residual ATC. The 12-month OS and PFS for patients with pathologic ATC CR were both 100%. The 12-month OS and PFS for patients with any ATC in their surgical specimen were 83.3% [CI 62.6–100] and 61.5% [CI 35.1–88], respectively.

## Discussion

This study represents the largest single institution cohort of consecutive BRAF^V600E^-mutated ATC patients in the published literature. The 32 patients in the neoadjuvant + surgery group had markedly reduced tumor burden, resulting in significantly improved ability to resect previously inoperable tumors, as well as decreased extent of surgical resection involving vital structures. For these patients, 24-month OS and PFS were 80.3% and 62.2%, respectively. In comparison, in a phase II basket trial for patients who received BRAF/MEK inhibitors, the 24-month OS and PFS were 31.5% and 27%, respectively.^9^ This suggests that surgery may play a beneficial role in OS and PFS for patients who receive BRAF-directed therapy. However, 97% of patients in the basket trial were stage IVC, while in our neoadjuvant + surgery group 62.5% were stage IVC. Nevertheless, we did not find that stage (IVB vs. IVC) demonstrated a significant impact on OS or PFS for patients in the neoadjuvant + surgery group. Furthermore, even with a median follow-up of ∼3 years, median OS was not reached in this cohort, while in a comparative study where patients with ATC received traditional trimodality treatment of surgery and chemoradiation, the median OS was 22.1 months.^15^ This difference in median OS suggests that the addition of BRAF-directed therapy with surgery ± chemoradiation may allow for superior survival as compared with traditional trimodality therapy.

Subgroup analysis suggested that age and stage did not alter OS or PFS for patients who had surgery after BRAF-directed therapy. As the majority of patients present with IVC disease or develop distant metastasis during the course of disease progression, our data suggest that neoadjuvant BRAF-directed therapy results in a systemic reduction in tumor burden to allow for surgical resection of primary disease. Most notably, subgroup analysis suggested that OS and PFS was significantly better for patients with pathologic ATC CR, thereby having potential to guide therapeutic decision making with further study.^16–18^ The pathologic data from this study also support the prevailing theory that the majority of BRAF^V600E^-mutated ATC arises from pre-existing PTC.^16–18^

Most patients (70%) who were started on BRAF-directed therapy with intent for surgical resection ultimately underwent surgery. In addition to BRAF-directed therapy, 84% of patients were also treated with upfront immunotherapy, typically started about 4 weeks after BRAF-directed therapy. The rationale of adding immunotherapy is owing to the high percentage of PD-L1 expression in ATC, preclinical research demonstrating benefit of immunotherapy to BRAF inhibition, and retrospective data suggesting benefit of adding immunotherapy to BRAF-directed therapy in BRAF-mutated ATC.^10,19,20^ We have recently reported that immunotherapy and surgery are independently associated with improved OS and PFS in BRAF-mutated ATC patients treated with BRAF-directed therapy.^10^ We believe that once patients reach a plateau in their treatment response, typically after about 3 months of therapy, surgical evaluation should be considered, to mitigate the risk of resistance and tumor progression.

At this time, BRAF-directed therapy is generally continued after surgery indefinitely, even among patients without structural evidence of disease. BRAF and MEK inhibitors are typically restarted within one week of surgery. As such, unlike the role of systemic therapy in other surgical diseases, BRAF-directed therapy may be thought of as the primary treatment modality for BRAF^V600E^-mutated ATC, rather than as a temporary neoadjuvant or adjuvant treatment. Similarly, surgery is not the “de facto” upfront treatment for most patients, as the majority present with stage IVC disease or ultimately develop distant metastasis. However, as suggested by the results of this study, the addition of surgery to BRAF-directed therapy is associated with improved OS and PFS.

Limitations of this study include the retrospective design, and the inability to definitively compare the three groups of patients presented herein owing to selection bias. While the main focus of this study is the 32 patients who underwent neoadjuvant + surgery, the other 2 groups of patients (patients who had upfront surgery and patients who had BRAF/MEK inhibitor without surgery) were presented herein to provide broad context, rather than as a direct comparison between the groups. Given the retrospective nature, selection bias and differences among these groups are expected. An additional limitation is the use of an unvalidated TNMC scoring system to assess for the effect of neoadjuvant therapy on surgical morbidity (as no validated scoring system is available for this purpose). The TNMC scoring system is currently being utilized in a number of multicenter advanced thyroid cancer trials (NCT04759911, NCT04675710), with a plan for clinical validation. Finally, this study focuses only on BRAF^V600E^-mutated patients, who account for only ∼30% of ATC. There are ongoing clinical trials to study targeted therapy and immunotherapy approaches for non-BRAF-mutated ATC patients (NCT04171622).

In summary, BRAF-mutated ATC patients who receive neoadjuvant BRAF-directed therapy followed by surgery have 24-month OS of 80%, which is especially noteworthy as nearly two-thirds of patients in this cohort had stage IVC disease. This OS compares favorably with both recent phase II clinical trial patients treated with BRAF-directed therapy without surgery,^9^ and recent retrospective cohort patients treated with upfront surgery followed by chemoradiation therapy.^15^ Of note, current American Thyroid Association (ATA) guidelines recommend patients with resectable ATC (IVA and IVB), irrespective of BRAF^V600E^ status, to undergo trimodally treatment with surgery followed by definitive chemoradiotherapy, while only patients with unresectable IVB and IVC disease are recommended for upfront BRAF-directed therapy.^21^ Given the significant primary tumor response with improved surgical resectability and decreased morbidity, coupled with favorable OS and PFS, we advocate that all patients with stage IVB and IVC BRAF^V600E^-mutated ATC be treated with neoadjuvant BRAF-directed therapy with intent for surgical consolidation of residual disease. Ongoing clinical trials (NCT04675710, NCT03181100) will continue to elucidate the roles of immunotherapy, surgery, and radiation therapy as adjuvant therapies to BRAF-direct therapy in patients with BRAF^V600E^-mutated ATC.

## Supplementary Material

Supplemental data

Supplemental data

Supplemental data

Supplemental data
