# Supplementary figures and images for: Surgery After BRAF-Directed Therapy Is Associated with Improved Survival in BRAFV600E Mutant Anaplastic Thyroid Cancer: A Single-Center Retrospective Cohort Study
Source: Thyroid. 2023 Apr 10;33(4):484–91. doi: 10.1089/thy.2022.0504 (PMC10122263; doi:10.1089/thy.2022.0504)

**Supplementary Table 2: Thyroid Neck Morbidity Complexity (TNMC) scoring system**


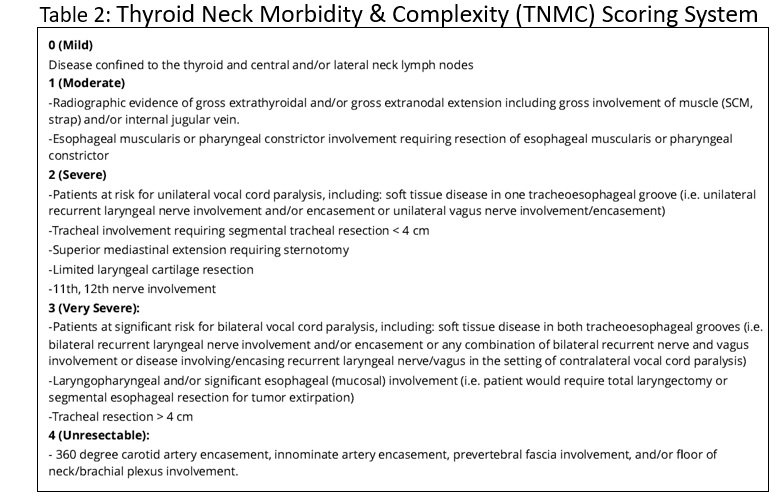

Supplement: Supplemental data [file Supp_TableS2.docx]
