# Supplementary material for: Surgery After BRAF-Directed Therapy Is Associated with Improved Survival in BRAFV600E Mutant Anaplastic Thyroid Cancer: A Single-Center Retrospective Cohort Study
Source: Thyroid. 2023 Apr 10;33(4):484–91. doi: 10.1089/thy.2022.0504 (PMC10122263; doi:10.1089/thy.2022.0504)
Supplement: Supplemental data [file Supp_TableS1.docx]

**Supplementary Table 1: Statistical testing of demographics and clinical characteristics**

|  | Comparison | P-value | Statistical methods |
| --- | --- | --- | --- |
| Age | Among 3 groups | 0.0393 | ANOVA |
| Sex | 3 groups | 0.6079 | Chi square test |
| AJCC Stage | 3 groups | 0.1765 | Chi square test |
| Immunotherapy | 3 groups | 0.1699 | Chi square test |
| Post-op XRT | 2 groups | 0.015 | Fisher exact test |
