# Supplementary material for: Surgery After BRAF-Directed Therapy Is Associated with Improved Survival in BRAFV600E Mutant Anaplastic Thyroid Cancer: A Single-Center Retrospective Cohort Study
Source: Thyroid. 2023 Apr 10;33(4):484–91. doi: 10.1089/thy.2022.0504 (PMC10122263; doi:10.1089/thy.2022.0504)
Supplement: Supplemental data [file Supp_TableS3.docx]

**Supplementary Table 3: Complications in the neoadjuvant+surgery group (n=32)**

| **Neoadjuvant BRAF-Directed Therapy** | |
| --- | --- |
| **Complication** | **Number of patients** |
| Fever/bactermia/sepsis | 5 |
| Epithelial reaction (photosensitivity, rash, mucositis) | 3 |
| Hepatotoxicity | 2 |
| Atrial fibrillation | 2 |
| Rhabomyolysis | 1 |
| Electrolyte disturbances | 1 |
| **Surgical Complications** | |
| **Complication** | **Number of patients** |
| Neck abscess | 2 |
| Ischemic stroke post-operative day 1 | 1 |
| Hypothyrodism readmission | 1 |
