# Supplementary material for: Surgery After BRAF-Directed Therapy Is Associated with Improved Survival in BRAFV600E Mutant Anaplastic Thyroid Cancer: A Single-Center Retrospective Cohort Study
Source: Thyroid. 2023 Apr 10;33(4):484–91. doi: 10.1089/thy.2022.0504 (PMC10122263; doi:10.1089/thy.2022.0504)
Supplement: Supplemental data [file Supp_TableS4.docx]

**Supplementary table 4: Subgroup analysis (patients in the neoadjuvant + surgery group, N=32)**

|  |  |  | OS | | | PFS | | |
| --- | --- | --- | --- | --- | --- | --- | --- | --- |
|  |  | N | Log-rank P | Univariate P* | Multivariate P** | Log-rank P | Univariate P* | Multivariate P** |
| Stage | IVB | 12 | 0.18 | 0.19 | 0.08 | 0.89 | 0.89 | 0.90 |
|  | IVC | 20 |  |  |  |  |  |  |
| TNMC | 0 | 22 | 0.73 | 0.74 | 0.82 | 0.77 | 0.77 | 0.69 |
|  | >0 | 10 |  |  |  |  |  |  |
| Time to surgery | <135.5 d | 16 | 0.91 | 0.91 | 0.66 | 0.71 | 0.71 | 0.82 |
|  | ≥135.5 d | 16 |  |  |  |  |  |  |
| Time to surgery | Below 95%CI | 23 | 0.68 | 0.69 | 0.20 | 0.94 | 0.93 | 0.89 |
|  | Above 95%CI | 9 |  |  |  |  |  |  |

* Univariate Cox proportional hazard regression model

** Multivariate Cox proportional hazard regression model, adjusted for age and stage
